# Editorial: The roles and mechanisms of hepatokines, adipokines and myokines in the development of non-alcoholic fatty liver disease (NAFLD)

**DOI:** 10.3389/fendo.2022.1074842

**Published:** 2022-12-16

**Authors:** Yan Lu

**Affiliations:** Department of Endocrinology and Metabolism, Zhongshan Hospital, Fudan University, Shanghai, China

**Keywords:** adipokine, hepatokines, myokines, nonalcoholic fatty live disease, liver

Nonalcoholic fatty liver disease (NAFLD), which is characterized by excessive triglyceride (TG) accumulation in hepatocytes, have become a serious health problem worldwide (1). Due to the changes of life style, such as overnutrition and lack of exercise, the prevalence of NAFLD is increasing year by year. NAFLD can further progress to nonalcoholic steatohepatitis (NASH) liver fibrosis, and hepatocellular carcinoma (2). Besides, both human studies and animal experiments have shown that NAFLD is strongly associated with systemic metabolic disorders, including hyperglycemia, insulin resistance and dyslipidemia (3). However, until now, there is no FDA-approved drugs for treating NAFLD and NASH. Therefore, a better understanding the molecular mechanisms of NAFLD and NASH remains urgent.

Decades of studies have demonstrated that cytokines derived from metabolic organs, including liver, adipose tissues and skeletal muscles, play crucial role in the regulation of hepatic and systemic lipid homeostasis (4). These organokines, termed as hepatokines, adipokines and myokines, could modulate lipid synthesis, oxidation and transport through autocrine, paracrine and endocrine manners. For instance, the role and mechanisms of hepatokine FGF21, adipokine adiponectin, and myokine Irisin in the development of NAFLD have been well-acknowledged (5–8). More importantly, these studies have provided potential therapeutic targets for the treatment of NAFLD and NASH.

Therefore, at this stage, we set up a Research Topic entitled “The Roles and Mechanisms of Hepatokines, Adipokines and Myokines in the Development of Non-Alcoholic Fatty Liver Disease (NAFLD)” in the Frontiers in Endocrinology. Through this Research Topic, we aimed to further establish the roles of hepatokines, adipokines and myokines in NAFLD and NASH. In this collection, Guo et al., identified dysregulated myokines in the development of NAFLD and NASH through comprehensive transcriptome profiling. Mao et al., identified adipokines and hepatokines associated with high-salt-diet in mice. Wang et al., analyzed the relationship between circulating Ism1 and diabetes and diabetes-associated NAFLD. Gao et al., provided a novel view on endoplasmic reticulum-related and secretome gene in NAFLD progression.

Overall, these studies together identified some new cytokines associated with NAFLD pathogenesis, which further strengthened our understanding of metabolic liver disease. However, intensive work is still required, such as investigations into the roles and mechanisms of these cytokines through genetic models, translational studies in human subjects, and screening potential therapeutic target for treatment. We hope that more and more studies on this Research Topic would help us better understand the molecular mechanisms of NAFLD development and identify more therapeutic targets for its treatment.

## Author contributions

The author confirms being the sole contributor of this work and has approved it for publication.
